# Bond cleavage of lignin model compounds into aromatic monomers using supported metal catalysts in supercritical water

**DOI:** 10.1038/srep46172

**Published:** 2017-04-07

**Authors:** Aritomo Yamaguchi, Naoki Mimura, Masayuki Shirai, Osamu Sato

**Affiliations:** 1 Research Institute for Chemical Process Technology, National Institute of Advanced Industrial Science and Technology (AIST), 4-2-1 Nigatake, Miyagino, Sendai 983-8551, Japan; 2 JST, PRESTO, 4-2-1 Nigatake, Miyagino, Sendai 983-8551, Japan; 3 Department of Chemistry and Biological Sciences, Faculty of Science and Engineering, Iwate University, Ueda 4-3-5, Morioka, Iwate 020-8551, Japan

## Abstract

More efficient use of lignin carbon is necessary for carbon-efficient utilization of lignocellulosic biomass. Conversion of lignin into valuable aromatic compounds requires the cleavage of C–O ether bonds and C–C bonds between lignin monomer units. The catalytic cleavage of C–O bonds is still challenging, and cleavage of C–C bonds is even more difficult. Here, we report cleavage of the aromatic C–O bonds in lignin model compounds using supported metal catalysts in supercritical water without adding hydrogen gas and without causing hydrogenation of the aromatic rings. The cleavage of the C–C bond in bibenzyl was also achieved with Rh/C as a catalyst. Use of this technique may greatly facilitate the conversion of lignin into valuable aromatic compounds.

Lignocellulosic biomass has attracted attention for use as a renewable carbon resource for a sustainable society^1^,^2^. Lignocellulose, which is inedible for humans, is the most abundant form of biomass and is therefore a promising alternative to fossil fuels^3^. Lignocellulose consists of cellulose (40–50%), hemicellulose (20–30%), and lignin (10–35%). Cellulose and hemicellulose are polysaccharides that can be converted into valuable products via saccharification and fermentation or via chemical processes^4^,^5^,^6^. We have reported that cellulose and hemicellulose in lignocellulose can be directly converted without delignification into sugar alcohols such as sorbitol, mannitol, and xylitol via supported metal catalysts with hydrogen gas; lignin remains as a solid after the reaction^7^,^8^. Conversion of this lignin into valuable products is thus important to achieve carbon-efficient use of lignocellulosic biomass. Lignin is a complex, three-dimensional polymer of aromatic compounds such as *p*-coumaryl, coniferyl, and sinapyl alcohols, with cross-linking via C–O–C ether bonds and C–C bonds (Fig. 1). Research on lignin depolymerization into valuable aromatic chemicals has grown rapidly in recent years^9^,^10^. Many researchers have reported depolymerizing lignin into aromatic products. Three main methods have been used^9^,^10^: (i) depolymerization using acids or bases, (ii) oxidative depolymerization, and (iii) reductive depolymerization. Depolymerization with acids^11^,^12^,^13^ or bases^14^,^15^,^16^ has traditionally been used to remove lignin for kraft pulping rather than to make use of the lignin. In this case, a complex aromatic mixture of phenols with alkyl and methoxy groups, as well as char, is formed from the lignin. The compounds produced from lignin using acids or bases must be separated or upgraded to homogeneous products, a challenging process. Furthermore, the use of acids or bases requires neutralization and purification and accelerates corrosion of the reactor walls. Oxidative depolymerization of lignin has also been one of the methods used to produce aromatic compounds from lignin^9^,^10^. Oxidative depolymerization tends to produce complex aromatic compounds with multiple functional groups. The advantage of oxidative depolymerization is that it is possible to cleave the C–C bonds between lignin monomers^17^,^18^,^19^,^20^. However, the aromatic compounds with multiple functional groups that result from oxidative reactions include a wide variety of products that must be separated or upgraded to homogeneous products. Conversely, because reductive reactions tend to remove the functional groups of the aromatic compounds produced from lignin, the products are simple phenols. The most promising method for cleaving the C–O bonds to selectively depolymerize lignin into aromatic monomers such as benzene and phenol is hydrogenolysis or hydrodeoxygenation with hydrogen gas. We therefore focused on hydrogenolysis of lignin model compounds in this report instead of oxidation or depolymerization using acids or bases. Several homogeneous catalysts have recently been investigated for the selective hydrogenolysis of aromatic C–O bonds^21^,^22^,^23^,^24^,^25^. Sergeev and Hartwig have reported that a nickel carbene complex catalyzes the selective hydrogenolysis of aromatic C–O bonds in Ar–OAr and Ar–OMe (Ar = aryl, Me = methyl) at 393 K with 0.1 MPa hydrogen gas using a strong base, *t*-BuONa (*t*-Bu = *tert-*butyl), and *m*-xylene as a solvent^23^. Homogeneous catalysts show high selectivity for the hydrogenolysis reactions of aromatic lignin model compounds under mild conditions; however, use of those catalysts is compromised by difficulties associated with product separation from the catalysts, stability and recyclability of the catalysts, and use of the catalysts for the hydrogenolysis of insoluble lignin. Some heterogeneous compounds have also been reported to catalyze the hydrogenolysis of aromatic lignin model compounds^26^,^27^,^28^,^29^; however, in those cases, the hydrogenolysis required hydrogen gas at high pressure, and hydrogenation of phenols resulted in the production of aliphatic hydrocarbons along with the products of hydrogenolysis.

Lignin is a polymer of aromatic compounds that have both C–O–C ether bonds and C–C bonds, the implication being that C–C bond cleavage is also important for the production of aromatic monomers from lignin. Cleavage of C–C bonds, however, is more difficult because of their high bond-dissociation energy^30^.

In this communication, we report cleavage of the aromatic C–O bonds in lignin model compounds by the use of supported metal catalysts in supercritical water without the addition of hydrogen gas; cleavage was achieved without causing hydrogenation of the aromatic ring. We selected 2-phenethyl phenyl ether and bibenzyl as model lignin compounds (Fig. 1); these compounds have C–O and C–C bonds characterized by β-O-4 and β-1 linkages, respectively, between the aromatic lignin units. We also found that the kinds of reactions used in this study could be applied to cleavage of the C–C bond in bibenzyl with Rh/C as a catalyst.

## Results and Discussion

Cleavage of the C–O bond in 2-phenethyl phenyl ether was carried out for 1 h in supercritical water at 673 K and a water density of 0.5 g cm^−3^ without hydrogen gas using Pd/C, Pt/C, Rh/C, or Ru/C as a catalyst or with no catalyst (Fig. 2). In the case of the Ru/C catalyst, 80 C% of 2-phenethyl phenyl ether was gasified into methane, carbon dioxide, and hydrogen, consistent with previous results that have shown that Ru/C catalyzes lignin gasification^31^. The activity of the catalysts for gasification decreased in the order Ru/C > Rh/C > Pt/C > Pd/C. An important point about the results of the study was the nature of the aromatic products. 2-Phenethyl phenyl ether could be converted into phenol (40.9 C% yield) and ethylbenzene (23.1 C% yield) using the Pd/C catalyst, the interesting indication being that hydrogenolysis of the C–O ether bonds in 2-phenethyl phenyl ether proceeded without hydrogen gas in supercritical water at 673 K. The hydrogenolysis reaction requires hydrogen gas or hydrogen atoms adsorbed on the surface of the catalyst metal. In this reaction, a part of the reactant compound was gasified into carbon dioxide, methane, ethane, and hydrogen (see Supplementary Table 1). Thus, hydrogen gas or surface-adsorbed hydrogen atoms, produced by the decomposition of the reactant, caused the hydrogenolysis reaction.

Hydrogenated products such as cyclohexane and cyclohexanol were not detected in this reaction system. Hydrogenation of the aromatic ring is thermodynamically unfavorable at 673 K during the equilibrium between aromatic compounds and hydrogenated compounds^32^. Valuable aromatic monomers could thus be obtained by the hydrogenolysis reaction without the hydrogenation reaction, an advantage of carrying out the reaction in supercritical water at 673 K. In addition, supercritical water acts like an organic solvent with a low dielectric constant and can eliminate transport limitation of reaction rates.

**Figure 1:**
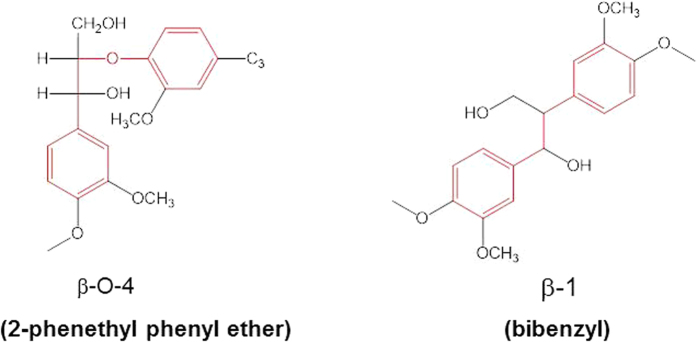
Linkages between aromatic monomers of lignin and lignin model compounds (red) examined in this study.

The β-O-4 bond in lignin contains functional groups Cα–OH and aromatic methoxy groups (Fig. 1). It has been reported that the Cα–OH is unstable in high-temperature water during the cleavage of β-O-4 bonds, in which case the cleavage of a C–O bond can proceed with a catalyst^33^,^34^. Under specific conditions, the Cα–OH group is dehydrogenated into a carbonyl group, and then aromatic products with a carbonyl group can be obtained. We plan to investigate the cleavage of lignin model compounds containing a Cα–OH in the future.

**Figure 2:**
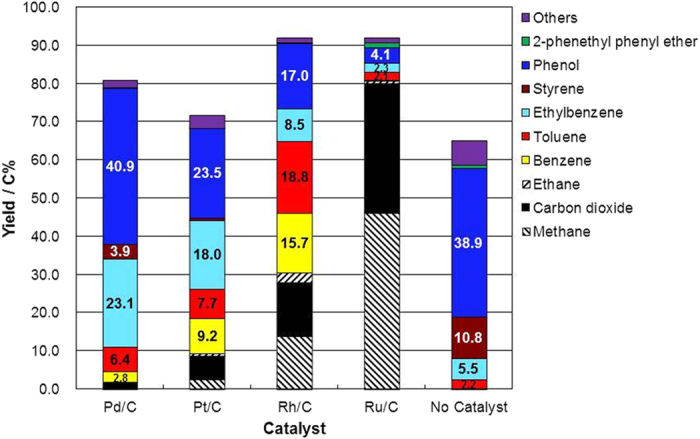
Product yield from 2-phenethyl phenyl ether after treatment for 1 h in supercritical water at 673 K and a water density of 0.5 g cm^−3^ with Pd/C, Pt/C, Rh/C, or Ru/C as a catalyst or with no catalyst.

Small yields of styrene, toluene, and benzene were also observed (Fig. 2). In the next section, we will explain how phenol and ethylbenzene were converted into benzene and toluene. That conversion can explain how benzene and toluene were produced from 2-phenethyl phenyl ether. Without catalysts, only a small amount of ethylbenzene was obtained, and gaseous products, including hydrogen gas, were not observed; the implication is that hydrogenolysis did not occur and that the stainless steel of the reactor wall did not show activity for the hydrogenolysis. Styrene, which can be obtained from the dehydration of 2-phenethyl alcohol, was obtained in water without catalysts. One possibility is that hydrolysis of 2-phenethyl phenyl ether into phenol and 2-phenethyl alcohol might occur without catalysts^35^,^36^. The other possibility is that homolytic cleavage of C–O occurred to form phenethyl and phenoxy radicals^37^, which produce styrene and phenol, respectively.

In reactions catalyzed by Pt/C and Rh/C, phenol and ethylbenzene were observed in the aromatic products, and the phenol yield was higher than the ethylbenzene yield, similar to the pattern observed with Pd/C. With Rh/C as a catalyst, the yields of benzene and toluene were higher than the yield of ethylbenzene.

**Figure 3:**
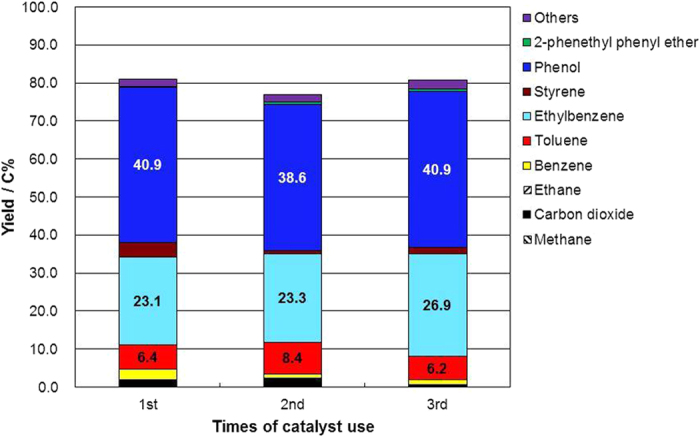
Recycling results for conversion of 2-phenethyl phenyl ether in supercritical water at 673 K for 1 h and a water density of 0.5 g cm^−3^ with Pd/C as a catalyst.

We investigated the possibility of reusing the heterogeneous catalysts Pd/C, Pt/C, and Rh/C by using them three times for the conversion of 2-phenethyl phenyl ether. The catalysts were recovered by filtration after the reaction and dried in an oven. The catalysts were then reused without any further treatment. The reactant 2-phenethyl phenyl ether was added each time, and the yields were calculated based on the amount of added reactant. The phenol yields with Pd/C were nearly unchanged; they varied from 40.9 C% (1st run) to 38.6 C% (2nd run) and 40.9 C% (3rd run) (Fig. 3), the indication being that Pd/C was not deactivated during the conversion of 2-phenethyl phenyl ether in supercritical water. In the case of Pt/C and Rh/C, the phenol yield increased gradually with recycling of the catalysts (see Supplementary Figs 1 and 2). The reason for the increase of phenol yield with catalyst recycling is still unclear; however, the stability and recyclability of the catalysts for the conversion of 2-phenethyl phenyl ether were experimentally demonstrated.

**Figure 4:**
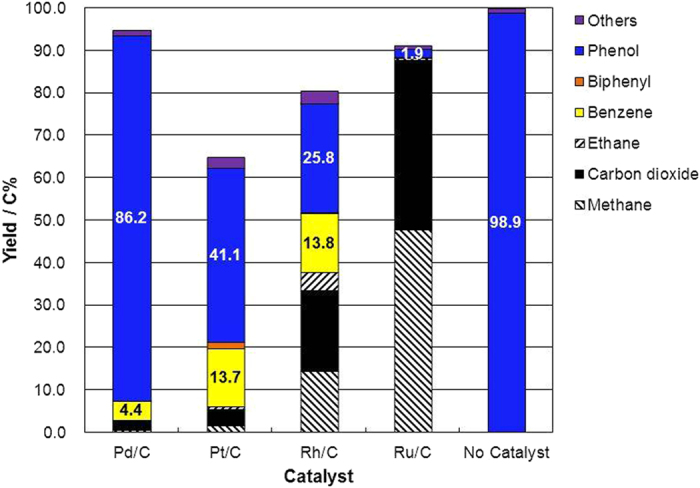
Product yield from phenol after treatment for 1 h in supercritical water at 673 K and a water density of 0.5 g cm^−3^ with Pd/C, Pt/C, Rh/C, or Ru/C as a catalyst or with no catalyst.

Decomposition of phenol was carried out for 1 h in supercritical water at 673 K using Pd/C, Pt/C, Rh/C, or Ru/C as a catalyst or with no catalyst (Fig. 4); the objective was to understand the distribution of products in the reaction of 2-phenethyl phenyl ether. Phenol was recovered from the reaction without a catalyst. Part of the phenol was converted into benzene by hydrodeoxygenation in the presence of Rh/C, Pt/C, and Pd/C. In that case, part of phenol was also gasified into carbon dioxide, methane, ethane, and hydrogen. Hydrogen gas was used in the hydrodeoxygenation of phenol into benzene. Part of the phenol obtained from 2-phenethyl phenyl ether must have been converted into benzene; thus, the yield of phenol from 2-phenethyl phenyl ether was low when the reaction was catalyzed by Rh/C and Pt/C (Fig. 2), which catalyzed the hydrodeoxygenation of phenol into benzene.

**Figure 5:**
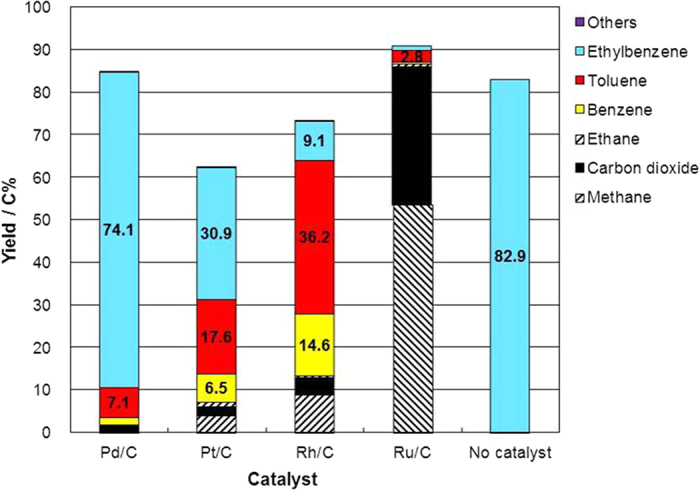
Product yield from ethylbenzene after treatment for 1 h in supercritical water at 673 K and a water density of 0.5 g cm^−3^ with Pd/C, Pt/C, Rh/C, or Ru/C as a catalyst or with no catalyst.

Decomposition of ethylbenzene was also carried out for 1 h in supercritical water at 673 K using Pd/C, Pt/C, Rh/C, or Ru/C as a catalyst or with no catalyst (Fig. 5). In the absence of a catalyst, ethylbenzene was recovered after the treatment in supercritical water at 673 K. In the presence of Pd/C, Pt/C, and Rh/C, toluene and benzene could be obtained from ethylbenzene, the indication being that these catalyzed the cleavage of the C–C bond. The yields of toluene and benzene from ethylbenzene conversion decreased in the order Rh/C > Pt/C > Pd/C. These results can explain the production of toluene and benzene from 2-phenethyl phenyl ether, especially in the case of Rh/C (Fig. 2). First, 2-phenethyl phenyl ether was converted into phenol and ethylbenzene by hydrogenolysis over the Rh/C catalyst; then ethylbenzene was converted into toluene and benzene. When Pd/C was the catalyst, the yields of toluene and benzene from ethylbenzene conversion were low (Fig. 5); thus, ethylbenzene could be obtained from 2-phenethyl phenyl ether rather than toluene and benzene (Fig. 2).

The time course of the yields of products from the conversion of 2-phenethyl phenyl ether was monitored with the catalysts Pd/C, Pt/C, and Rh/C (see Supplementary Figures 1, 2 and 3). The yields of phenol and ethylbenzene reached maximum values at a short reaction time (20 min) and subsequently decreased. Conversely, the yields of toluene and benzene increased with increasing reaction time. The indication is that ethylbenzene and phenol were converted into toluene and benzene, a conclusion that is consistent with our experimental results (Figs 4 and 5). The conversion of ethylbenzene and phenol was slow with Pd/C (Figs 4 and 5). The yields of phenol and ethylbenzene from 2-phenethyl phenyl ether thus decreased slightly with increasing reaction time (see Supplementary Figure 1). Time-course experiments could explain the proposed reaction mechanism; however, we cannot eliminate the possibility of direct conversion of 2-phenethyl phenyl to toluene and benzene at a reaction time less than 20 min. In this reaction system, it is difficult to correctly evaluate the activity at a reaction time of less than 20 min.

**Figure 6:**
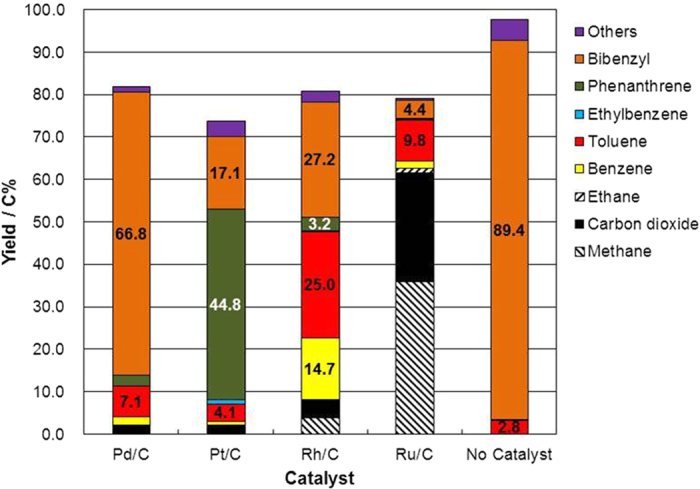
Product yield from bibenzyl after treatment for 1 h in supercritical water at 673 K and a water density of 0.5 g cm^−3^ with Pd/C, Pt/C, Rh/C, or Ru/C as a catalyst or with no catalyst.

The results of ethylbenzene decomposition indicated that the C–C bond could be cleaved using either Rh/C or Pt/C as a catalyst; we thus attempted to cleave the C–C bond of bibenzyl, a model lignin compound, using the supported metal catalysts in supercritical water (Fig. 6). Surprisingly, toluene and benzene could be obtained from bibenzyl using Rh/C, the indication being that the C–C bond in bibenzyl was cleaved. Cleavage of the C–C bonds in the linkages between the aromatic monomers of a model lignin compound has not been reported; this result can therefore open a new pathway for lignin decomposition research. Phenanthrene was obtained from bibenzyl, especially when Pt/C was the catalyst (Fig. 6), the indication being that Pt/C was an excellent catalyst for the dehydrogenation reaction. The Pd/C catalyst only slowly catalyzed the cleavage of the C–C bond in bibenzyl, the indication being that the species of metal was an important determinant of the behavior of lignin during its decomposition in supercritical water.

## Conclusion

We have demonstrated that lignin model compounds undergo some unique reactions in which the C–O and C–C bonds are cleaved without adding hydrogen gas and without causing hydrogenation of the aromatic ring. Cleavage of the C–O bonds in lignin model compounds has recently attracted considerable attention because the reactions can be used to decompose lignin into valuable aromatic monomers^9^,^10^. Homogeneous catalysts show high selectivity for bond cleavage via hydrogenolysis of aromatic lignin model compounds under mild conditions; however, those catalysts have limited applicability because of their relatively poor stability and recyclability. Heterogeneous catalysts are acknowledged to be the most likely candidates for decomposing lignin from a practical standpoint. Supported metal catalysts can catalyze the hydrogenolysis of aromatic lignin model compounds; however, hydrogenation of aromatic rings yielded aliphatic hydrocarbons among the products of hydrogenolysis. In this study, we were able to overcome this problem by using supported metal catalysts and supercritical water without hydrogen gas. Hydrogenolysis of C–O ether bonds in 2-phenethyl phenyl ether proceeded without hydrogen gas in supercritical water at 673 K using Pd/C, Pt/C, and Rh/C. Surprisingly, the C–C bond in bibenzyl was also cleaved in supercritical water at 673 K with Rh/C as a catalyst.

In conclusion, the cleavage of the C–O and C–C bonds in 2-phenethyl phenyl ether and bibenzyl has been achieved using supported metal catalysts in supercritical water without hydrogen gas. Application of this technique may greatly facilitate the use of lignin as a chemical feedstock.

## Methods

Charcoal-supported metal catalysts such as 5% Pd/C, 5% Pt/C, 5% Rh/C, and 5% Ru/C (denoted as Pd/C, Pt/C, Rh/C, and Ru/C) were purchased from Wako Pure Chemical Industries. Lignin model compounds were decomposed in a batch reactor made of stainless steel 316 tube with an inner volume of 6.0 cm^3^ in the similar method for lignin gasification in our papers^38^,^39^,^40^. In a typical catalytic reaction, 0.15 g of the catalyst, 0.10 g of lignin model compounds, and 3.0 g of water were added to the reactor. The reactor was sealed and purged with argon gas. The reactor was submerged in a molten-salt bath at 673 K for 1 h and then submerged in a water bath to quickly cool the contents of the reactor to ambient temperature after the reaction. The partial pressure of water in the reaction condition was 37.1 MPa and the water was in a supercritical state. The analyses of gas products were performed by gas chromatography (Shimadzu, GC-8A) with a thermal conductivity detector using a Shincarbon ST column. Liquid products in the reactor were recovered with tetrahydrofuran and filtered to separate solid catalysts from the liquid fraction. The analyses of liquid products were performed by gas chromatography (Agilent, HP-6890) with a flame ionization detector using a DB-WAX capillary column. The product yield was calculated using equation (1).


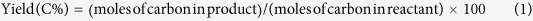


The yield of “others” was that of the identified aromatic compounds other than those shown in the figures.

## Additional Information

**How to cite this article**: Yamaguchi, A. *et al*. Bond cleavage of lignin model compounds into aromatic monomers using supported metal catalysts in supercritical water. *Sci. Rep.* **7**, 46172; doi: 10.1038/srep46172 (2017).

**Publisher's note:** Springer Nature remains neutral with regard to jurisdictional claims in published maps and institutional affiliations.

## Supplementary Material

Supplementary Information
