# Supplementary material for: Bond cleavage of lignin model compounds into aromatic monomers using supported metal catalysts in supercritical water
Source: Sci Rep. 2017 Apr 7;7:46172. doi: 10.1038/srep46172 (PMC5384005; doi:10.1038/srep46172)
Supplement: Supplementary Information [file srep46172-s1.doc]

Supplementary Information

**Bond cleavage of lignin model compounds into aromatic monomers using supported metal catalysts in supercritical water**

Aritomo Yamaguchi^1,2,*^, Naoki Mimura^1^, and Masayuki Shirai^1,3^, & Osamu Sato^1^

^1^ Research Institute for Chemical Process Technology, National Institute of Advanced Industrial Science and Technology (AIST), 4-2-1 Nigatake, Miyagino, Sendai 983-8551, Japan. ^2^ JST, PRESTO, 4-2-1 Nigatake, Miyagino, Sendai 983-8551, Japan.

^3^ Department of Chemistry and Biological Sciences, Faculty of Science and Engineering, Iwate University, Ueda 4-3-5, Morioka, Iwate 020-8551, Japan.

**Supplementary Figures:**

**
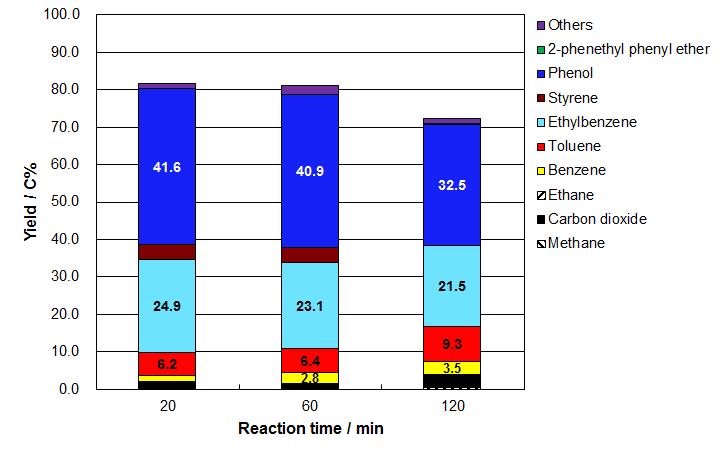
**

**Supplementary Figure 1** Product yield from 2-phenethyl phenyl ether as a function of elapsed time in supercritical water at 673 K and a water density of 0.5 g cm^–3^ with Pd/C as a catalyst.


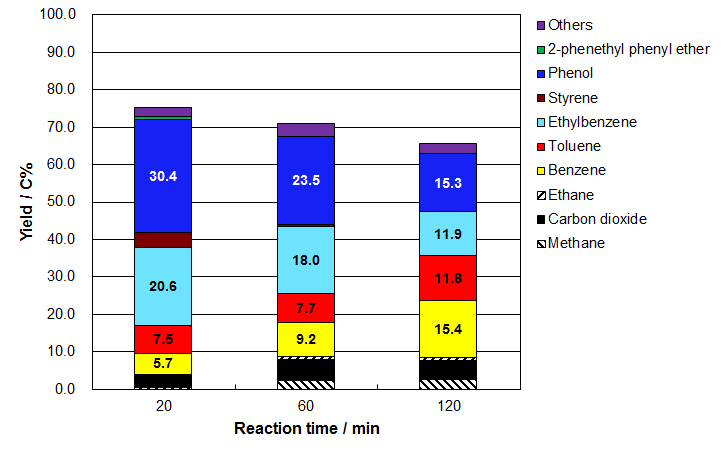


**Supplementary Figure 2** Product yield from 2-phenethyl phenyl ether as a function of elapsed time in supercritical water at 673 K and a water density of 0.5 g cm^–3^ with Pt/C as a catalyst.


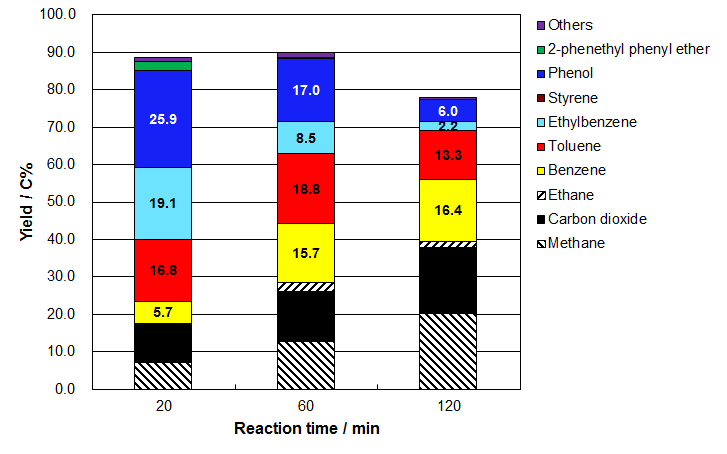


**Supplementary Figure 3** Product yield from 2-phenethyl phenyl ether as a function of elapsed time in supercritical water at 673 K and a water density of 0.5 g cm^–3^ with Rh/C as a catalyst.

**
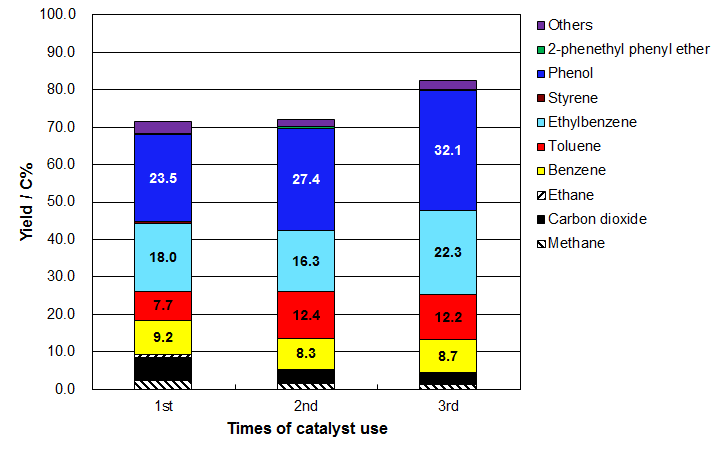
**

**Supplementary Figure 4** Recycling results for conversion of 2-phenethyl phenyl ether in supercritical water at 673 K for 1 h and a water density of 0.5 g cm^–3^ with Pt/C as a catalyst.

**
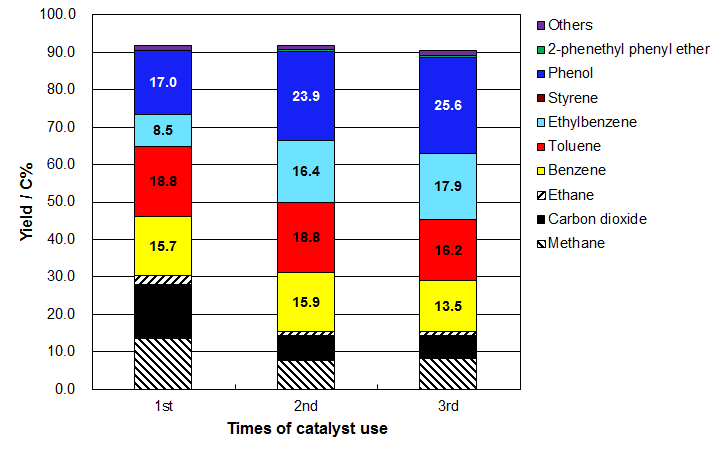
**

**Supplementary Figure 5** Recycling results for conversion of 2-phenethyl phenyl ether in supercritical water at 673 K for 1 h and a water density of 0.5 g cm^–3^ with Rh/C as a catalyst.

**Supplementary Table:**

**Supplementary Table 1** Composition of gases produced by decomposing 2-phenethyl phenyl ether via treatment for 1 h in supercritical water at 673 K and a water density of 0.5 g cm^–3^ with Pd/C, Pt/C, Rh/C, or Ru/C as a catalyst.

| **Catalyst** | **Gas composition (%)** | | | |
| --- | --- | --- | --- | --- |
|  | H_2_ | CH_4_ | CO_2_ | C_2_H_6_ |
| Pd/C | 1.2 | 8.1 | 90.7 | 0.0 |
| Pt/C | 2.2 | 27.1 | 67.2 | 3.5 |
| Rh/C | 2.4 | 45.8 | 47.7 | 4.2 |
| Ru/C | 3.3 | 55.6 | 40.7 | 0.4 |
